# Cooling immunochemotherapy and hematopoietic stem cell transplantation for refractory systemic epstein-barr virus-positive T-cell lymphoma in children: a single institution experience

**DOI:** 10.1007/s00277-026-06976-4

**Published:** 2026-04-18

**Authors:** Bo Kyung Kim, Kyung Taek Hong, Jung Yoon Choi, Hyun Jin Park, Hyoung Jin Kang

**Affiliations:** 1 Department of Pediatrics, Seoul National University College of Medicine, 101 Daehak-ro, Jongno-gu, Seoul, 03722 Korea; 2 Seoul National University Cancer Research Institute, Seoul, Korea

**Keywords:** Epstein-Barr virus-positive T-cell lymphoma, Hemophagocytic lymphohistiocytosis, Cooling immunochemotherapy, Hematopoietic stem cell transplantation

## Abstract

Systemic Epstein-Barr virus-positive T-cell lymphoma (SEBVTL) of childhood is a rare and aggressive disorder often associated with hemophagocytic lymphohistiocytosis (HLH) and multiorgan failure. We reviewed six pediatric SEBVTL cases treated at Seoul National University Children’s Hospital between 2017 and 2024. All patients presented with HLH and bone marrow involvement. Initial management included the HLH-2004 protocol and chemotherapy. Since December 2022, cooling immunochemotherapy (etoposide and cytarabine) was introduced as a bridge to hematopoietic stem cell transplantation (HSCT). Median age at diagnosis was 8.2 years, and median follow-up was 8.5 months. Three patients treated before 2022 died of progressive disease. Among three patients managed with cooling immunochemotherapy and HSCT, two survived and one died of engraftment failure. The 1-year overall and event-free survival rates were both 33.3%. These findings suggest cooling immunochemotherapy followed by HSCT may improve outcomes in refractory pediatric SEBVTL.

## Introduction

Pediatric systemic Epstein-Barr virus-positive T-cell lymphoma (SEBVTL) is a rare and highly aggressive malignancy primarily reported in East Asian countries [1]. It predominantly affects immunocompetent children and young adults and often presents with hemophagocytic lymphohistiocytosis (HLH), severe infections, and multiple organ failure, further complicating the disease course.

SEBVTL is considered part of the spectrum of Epstein-Barr virus (EBV)-associated T/NK-cell lymphoproliferative disorders, which includes chronic active EBV infection (CAEBV), hypersensitivity to mosquito bites, and extranodal NK/T-cell lymphoma [2]. These disorders share common pathogenetic mechanisms involving EBV-driven proliferation of T or NK cells. The disease is characterized by clonal T-cell proliferation driven by Epstein-Barr virus (EBV) infection, with neoplastic cells commonly expressing CD3 +, CD8 +, CD56-, and TIA-1 + markers. Epstein-Barr virus-encoded small RNA (EBER) in situ hybridization confirms EBV association, while T-cell receptor gene rearrangement establishes clonality [1, 3].

A major clinical challenge is that SEBVTL frequently presents with HLH, making it difficult to distinguish between EBV-associated HLH and overt lymphoma at initial presentation. In many cases, hyperinflammatory and neoplastic processes coexist. Although the precise mechanisms by which EBV infects T cells remain unclear, the clonal expansion of EBV-infected T cells and subsequent hyperactivation of the immune response can lead to hemophagocytic lymphohistiocytosis (HLH), which may result in a fatal clinical course [4–6]. Previous studies have also highlighted the overlap between EBV-driven lymphoproliferation and HLH, further complicating diagnosis and treatment decisions [7, 8].

Despite recent therapeutic advancements, prognosis remains poor, with most patients experiencing disease progression and fatal outcomes within months of onset [9]. In recent years, several cases have been reported in which patients with SEBVTL were treated with chemotherapy alone or in combination with hematopoietic stem cell transplantation [3, 10–12]. However, the number of reported cases worldwide remains limited and no standardized treatment has been established. Reports of refractory cases are scarce.

Despite the critical need for strategies that can suppress EBV-driven immune activation and stabilize disease activity prior to hematopoietic stem cell transplantation (HSCT), data on effective bridging approaches remain scarce. Here, we present our single-institution experience in treating pediatric and adolescent patients with SEBVTL, focusing on the outcomes of cooling immunochemotherapy followed by HSCT.

## Methods

### Study design

This study was a retrospective analysis of pediatric and adolescent patients diagnosed with SEBVTL of childhood at Seoul National University Children's Hospital between 2017 and 2024. Clinical data including demographics, laboratory findings, treatment regimens, and outcomes were collected and reviewed.

### Patient population

Patients included in this study were aged 18 years or younger at the time of diagnosis and met the diagnostic criteria for SEBVTL based on histopathologic, immunophenotypic, and molecular findings. All patients exhibited clinical features consistent with HLH. The extent of disease involvement, including in the bone marrow, liver, and lymph nodes, was assessed using imaging and biopsy. Clinical history and laboratory assessment were performed in all patients to exclude underlying immunodeficiency, and no evidence of primary immunodeficiency was identified. Genetic evaluation using an HLH gene panel was also conducted to assess for primary (familial) HLH.

### Treatment protocols

Patients with features of HLH were initially managed using an HLH-2004 regimen consisting of dexamethasone, etoposide, and cyclosporine. Upon confirmation of SEBVTL, chemotherapy was administered based on one or more of the following protocols: SMILE [13], 106B induction [14], ESCAP [15], and CHOP regimens [15] (Table 1). In December 2022, cooling immunochemotherapy consisting of etoposide and cytarabine was introduced as a bridging treatment before HSCT. Cooling immunochemotherapy was defined as continuous low-dose etoposide and cytarabine administered to suppress EBV-driven immune activation and stabilize disease activity prior to HSCT. Low-dose etoposide (30 mg/m^2^/day) and cytarabine (20 mg/m^2^/day) were continuously infused for 24 h for approximately 1.5 weeks before the initiation of conditioning [15]. This cooling immunochemotherapy was administered as a single cycle immediately prior to HSCT and was not repeated. The regimen was used as a bridging therapy to stabilize disease activity before transplantation. HSCT was performed using either haplo-identical donors (*n* = 2) or matched unrelated donors (*n* = 1) for engraftment, post-transplant complications, and closely monitored survival outcomes.

**Table 1:** Chemotherapy regimens for patients

Regimen	Drugs Included
SMILE	Methotrexate IV 2000 mg/m^2^, day 1 (Leucovorin rescue 15 mg/m^2^ IV every 6 h × 4, starting 24 h later) Ifosfamide 1500 mg/m^2^, day 2, 3, 4 (with 150 mL normal saline, Mesna co-administration) Dexamethasone 40 mg/m^2^, day 2, 3, 4 Etoposide 100 mg/m^2^ IV, day 2, 3, 4 L-Asparaginase 6000 U/m^2^, day 5, 7, 9, 11, 13, 15, 17 (total 7 doses) Intrathecal Cytarabine + Hydrocortisone, day 1
106B induction	Prednisone 60 mg/m^2^/day, day 1–28 (every 8 h) Vincristine 1.5 mg/m^2^, day 1, 8, 15, 22 L-Asparaginase 6000 U/m^2^, day 3, 6, 9, 12, 15, 18 (total 6 doses) Cyclophosphamide 1200 mg/m^2^, day 1 (with equal dose of Mesna) Daunorubicin 60 mg/m^2^, day 1, 8 Intrathecal Cytarabine + Hydrocortisone, day 1 Intrathecal Methotrexate + Hydrocortisone, day 15
ESCAP	Etoposide 150 mg/m^2^, day 1 High-dose Cytarabine 1500 mg/m^2^ every 12 h, day 1–4 T-FT Eye Drops (Flarex Ophthalmic Solution) 4 times/day, continued for 48 h after completion of Ara-C L-Asparaginase 6000 U/m^2^, day 5–9 Methylprednisolone 62.5 mg/m^2^ every 12 h, day 1–4 Prednisone 30 mg/m^2^/day, day 5–8
CHOP	Prednisone 50 mg/m^2^/day, day 1–5 Cyclophosphamide 750 mg/m^2^, day 1 (with equal dose of Mesna, administered at 0, 3, and 6 h) Adriamycin 50 mg/m^2^, day 1 Vincristine 1.5 mg/m^2^ (max. 2 mg), day 1 Intrathecal Cytarabine + Hydrocortisone, day 1

### Statistical analysis

The primary outcomes measured in this study were overall survival (OS) and event-free survival (EFS) at one year. Secondary outcomes included HLH recurrence, graft-versus-host disease (GVHD), and treatment-related mortality. Kaplan–Meier survival analysis was used to estimate OS and EFS, while descriptive statistics were used to summarize patient characteristics and treatment outcomes. Statistical comparisons were performed between patients treated before and after the introduction of cooling immunochemotherapy in December 2022.

### Ethics statement

This retrospective study was approved by the Institutional Review Board of Seoul National University Hospital (H-2509–083–1676).

## Results

### Patient characteristics

Six pediatric patients were included in this study. Patient characteristics are summarized in Table 2. The median age at diagnosis was 9.8 years (range, 5.5–17.1), with an equal distribution of male (*n* = 3) and female (*n* = 3) patients. Histologically, five patients (83%) were diagnosed with SEBVTL, while one patient (17%) was diagnosed with extranodal NK/T-cell lymphoma involving the bone marrow. All patients demonstrated bone marrow involvement with additional extranodal sites, including the cervical lymph nodes, liver, spleen, and small bowel.

**Table 2:** Characteristics of patients

Characteristics	N(%)
Sex	
Male	3 (50)
Female	3 (50)
Histology	
SEBVTL	6 (100)
Tumor location	
BM + Others (LN, liver, spleen, small bowel)	5(83)
BM	1 (17)
HLH(clinical diagnosis)	
Yes	6 (100)
No	0
HLH-2004 criteria ≥ 5	
Yes	5 (83)
No	1 (17)

*BM*, Bone marrow; *LN*, Lymph node; *HLH*, Hemophagocytic lymphohistiocytosis. HLH diagnosis was based on HLH-2004 criteria and clinical judgment

At initial presentation, all patients exhibited systemic inflammatory features consistent with HLH, including persistent fever, cytopenia, hyperferritinemia, and elevated EBV viral load. In addition to bone marrow involvement, extranodal disease was commonly observed, including cervical lymphadenopathy, hepatosplenomegaly, and gastrointestinal involvement such as small bowel lesions (Fig. 2). These findings indicated disseminated EBV-associated lymphoproliferative disease at diagnosis.

Immunophenotyping revealed consistent expression of CD3 and CD8. CD56 expression was negative in most cases, except for the patient diagnosed with NK/T-cell lymphoma, who showed CD56 positivity. T-cell receptor gene rearrangement was confirmed in all evaluated cases (5/5). EBER in situ hybridization was positive in all patients, and the quantitative EBV DNA titers in the whole blood ranged from 69,734 to 11,309,491 copies/mL (median: 3,340,204 copies/mL). The number of HLH-2004 criteria fulfilled at presentation ranged from 4 to 6. Five patients fulfilled at least 5 of 8 criteria, whereas one patient fulfilled 4 criteria but was clinically considered to have HLH based on a highly suggestive presentation in the context of EBV-driven lymphoproliferative disease. Histopathologic findings and EBV titers are summarized in Table 3.

**Table 3:** Histopathologic findings & EBV titer

Case	Histology	Site	CD3	CD8	CD56	TCRrearrangement	EBER	EBV-DNA (copies/mL whole blood)
1	SEBVTL	BM, cervical LN	+	+	-	+	+	661,440
2	SEBVTL	BM, Cervical LN, small bowel	+	+	-	+	+	69,734
3	SEBVTL	BM	+	+	-	+	+	11,309,491
4	extranodal NK/T cell lymphoma	BM, liver, spleen	+	+	+	+	+	5,909,023
5	SEBVTL	BM, liver	+	+	-	+	+	3,000,360
6	SEBVTL	BM, cervical LN	+	+	Not assessed	+	+	3,680,048

*SEBVTL*, Systemic epstein-BarrVirus-positive T-cell lymphoma; *BM*, Bone marrow; *LN*, Lymph node; *HLH*, Hemophagocytic lymphohistiocytosis

Bone marrow findings were heterogeneous across patients. Hemophagocytic histiocytes consistent with HLH were observed in several cases. In addition, varying degrees of infiltration by CD3 +/CD8 + EBV-positive T cells were identified, supporting bone marrow involvement by EBV-associated T-cell lymphoproliferative disease. The degree of marrow cellularity ranged from hypocellular to normocellular, and only a small number of atypical lymphoid cells were observed. No cases demonstrated blast proliferation sufficient to meet criteria for acute leukemia.

Genetic evaluation using an HLH gene panel was performed in all patients, and no pathogenic variants associated with familial HLH were identified.

### Patient outcomes

All patients initially received HLH-directed therapy, including HLH-2004-based regimens, at diagnosis due to the presence of hyperinflammatory features. However, as disease progression or insufficient response was observed, lymphoma-directed chemotherapy was subsequently administered. Lymphoma-directed regimens included SMILE, ESCAP, CHOP, DECAL, and 106B induction therapy, selected according to clinical status and treatment response. Despite these treatments, disease control was often inadequate, necessitating further therapeutic strategies. Individual treatment courses are summarized in Table 4. Three patients (Cases 1–3) subsequently underwent allogeneic HSCT, whereas the remaining three patients (Cases 4–6) were unable to receive HSCT due to disease progression or treatment-related complications.

**Table 4:** Treatment course, HSCT characteristics, and outcomes of patients

Case	Gender	Age (year)	Treatment (number of cycle)	Cooling immunochemotherapy	HSCT	Pre-HSCT disease status	Pre-HSCT HLH status	Donor type	Conditioning regimen/GVHD prophylaxis	EBV-DNA before HSCT (copies/mL whole blood)	EBV-DNA after HSCT (copies/mL whole blood)	EFS (month)	OS (month)	Survival	Cause of death
1	Female	9.8	HLH 2004 (-VP) (3) SMILE (1) ESCAP (1) Ruxolitinib	etoposide + cytarabine	Yes	LN (CR); BM (SD)	Resolved	Haplo-identical donor (mother)	TBI/Flu + PTCy	304,203	Not assessed	7.9	7.9	Died	Engraftment failure, atypical pneumonia
2	Female	12.5	HLH 2004(-VP) SMILE(3)	etoposide + cytarabine	Yes	LN/SB (CR); BM (NA)	Resolved	Matched unrelated donor	Bu/Flu/VP16 + ATG	69,450	694	23.3	23.3	Alive	-
3	Female	6.5	HLH 2004 (-VP) SMILE (1) CHOP (1)	etoposide + cytarabine	Yes	BM (SD)	Resolved	Haplo-identical donor (father)	Flu/Mel/Cy + PTCy	5,155,982	0	27.0	27.0	Alive	-
4	Male	5.5	HLH 2004 (1) RT (whole liver, 1980 cGy) 106B induction (1)	-	No	-		-	-		-	1.2	1.2	Died	HLH, multi-organ failure
5	Male	5.8	HLH 2004 (3) 106B induction (1) SMILE (1) DECAL (1)	-	No	-		-	-		-	9.1	9.1	Died	Lymphoma progression, HLH
6	Male	17.1	HLH 2004 (3) CHOP (1) ESCAP (2)	-	No	-		-	-		-	5.3	5.3	Died	HLH, multi-organ

*HSCT*, Hematopoietic stem cell transplantation; *HLH*, Hemophagocytic lymphohistiocytosis; *GVHD*, Graft-versus-host disease; *EFS*, Event-free survival; *OS*, Overall survival; *TBI*, Total body irradiation; Flu, fludarabine; *Bu*, Busulfan; *VP16*, Etoposide; Mel, melphalan; *Cy*, Cyclophosphamide; *ATG*, Anti-thymocyte globulin; *PTCy*, Post-transplant cyclophosphamide; *NA*, Not assessed; *LN*, Lymph node; *SB*, Small bowel; *BM*, Bone marrow; *CR*, Complete response; *SD*, Stable disease

**Fig. 1:**
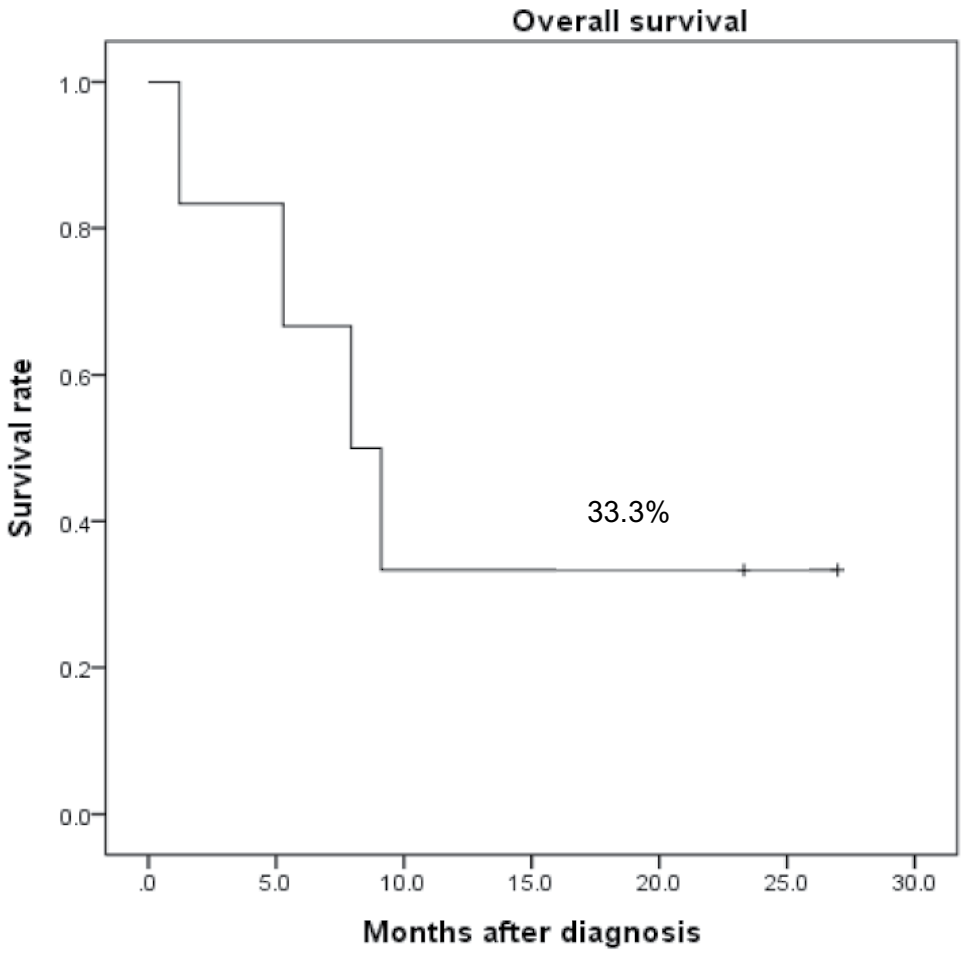
Overall survival of the patients

The 1-year OS and EFS rates were 33.3% (Fig. 1). Among the six patients, three diagnosed before December 2022 experienced uncontrolled disease progression and died from multiorgan failure. Three patients diagnosed after 2022 underwent cooling immunochemotherapy followed by allogeneic HSCT (two from haploidentical donors and one from a matched unrelated donor), resulting in two survivors. However, one patient experienced engraftment failure and died after the second HSCT (Table 4).

**Fig. 2:**
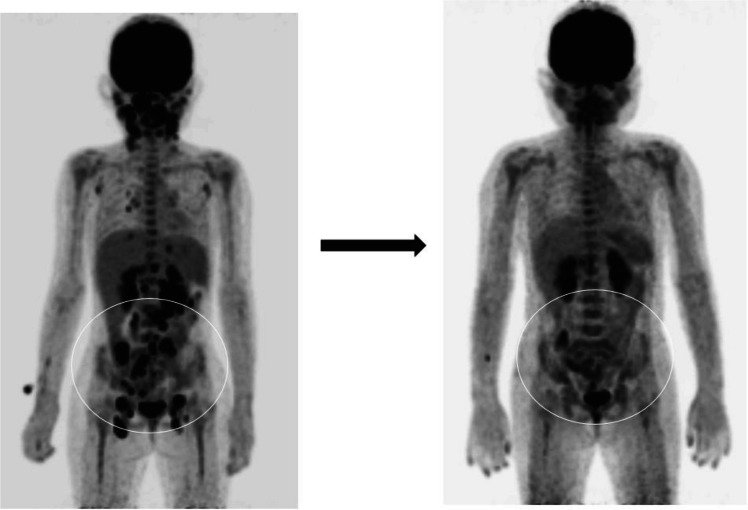
Representative PET–CT image showing increased fluorodeoxyglucose uptake in the small bowel. The lesion is indicated by a circle

Among the two surviving patients who underwent HSCT, both developed grade II acute GVHD, which was clinically tolerable and successfully managed with immunosuppressive therapy. One patient who underwent haploidentical HSCT developed hypotension and fever shortly after stem cell infusion, suspected to be due to an alloimmune reaction or systemic inflammatory response syndrome, and required temporary transfer to the intensive care unit. The patient improved after treatment with post-transplant cyclophosphamide and intravenous methylprednisolone. During follow-up of approximately two years, both patients have remained alive without significant long-term transplant-related complications (Fig. 2).

### Mortality

Among the four patients who died, one (Patient 1) underwent haploidentical HSCT following cooling immunochemotherapy but experienced graft failure after two transplant attempts and died due to multiorgan failure and infection. The remaining three patients (Patients 4, 5, and 6) received intensive chemotherapy regimens, including the HLH-2004 protocol and multiple chemotherapy regimens, but failed to achieve sufficient disease control to proceed with HSCT. All three patients died from complications related to uncontrolled HLH or disease progression, including hypovolemic shock, sepsis, and multiorgan failure.

## Discussion

In this study, we analyzed six pediatric patients diagnosed with SEBVTL, a rare and aggressive lymphoproliferative disorder, with limited treatment data. All patients presented with features of HLH and high EBV viremia at diagnosis.

Among the six patients, three diagnosed before December 2022 experienced rapid disease progression and died without undergoing HSCT. In response to these unfavorable outcomes, cooling immunochemotherapy consisting of etoposide and cytarabine was introduced at our institution in December 2022 as a bridging treatment prior to HSCT. This strategy was adopted based on previously reported approaches using continuous low-dose etoposide and cytarabine to stabilize EBV-driven disease activity and suppress immune activation [15].

Following the introduction of this strategy, three subsequent patients underwent allogeneic HSCT after cooling immunochemotherapy, of whom two achieved long-term remission. In contrast, one patient died due to graft failure. These findings suggest that cooling immunochemotherapy as a bridging strategy may facilitate successful HSCT and improve outcomes in pediatric SEBVTL, while the overall prognosis remains poor in the absence of timely disease control and definitive therapy.

Clinicians have explored various treatment strategies for SEBVTL due to its aggressive nature. The modified SMILE (mSMILE) chemotherapy regimen has shown promising efficacy, with a complete response rate of 62.5% in a cohort of pediatric patients [16]. HSCT has emerged as a potentially curative approach, particularly in patients who are refractory to conventional therapies [12]. However, the effectiveness of these treatment modalities is frequently compromised by rapid disease progression and severe complications, such as HLH, which substantially affect patient outcomes.

Given the fulminant course and high risk of early mortality in SEBVTL, prompt selection of effective chemotherapy is essential. In our cohort, the conventional regimens were insufficient for disease control in most patients. Based on the accumulating evidence that intensified chemotherapy regimens can suppress EBV viremia and reduce HLH activity [15], we used cooling immunochemotherapy as a bridging strategy to facilitate HSCT. For patients with adequate response to bridging therapy, a decision to proceed with HSCT was made, in line with previous studies supporting its curative potential in EBV-driven lymphoproliferative disorders [10, 16]. In our cohort, all patients treated before the adoption of cooling immunochemotherapy failed to achieve remission and died without transplantation. By contrast, survival was achieved in patients who received cooling chemotherapy as a bridging strategy and successfully underwent HSCT.

This study had several limitations. First, this was a retrospective, single-institutional case study with a small sample size, which limited the generalizability of the findings. Second, owing to the rarity and clinical heterogeneity of SEBVTL, treatment decisions were not uniform across patients, and potential confounding factors could not be fully controlled. Third, the follow-up period was relatively short in some patients, particularly those who underwent recent transplantation, limiting our ability to assess long-term outcomes such as late relapses or transplant-related complications.

Despite these limitations, our findings provide early evidence supporting the feasibility and potential benefits of cooling immunochemotherapy as a bridging strategy to facilitate HSCT in pediatric SEBVTL. Prospective multicenter studies with larger patient cohorts and standardized treatment approaches are warranted to validate these observations and optimize therapeutic strategies for this highly fatal disease.

## Data Availability

The study data is not publicly available to respect participant confidentiality. Requests for sharing of deidentified data should be directed to the corresponding author.
